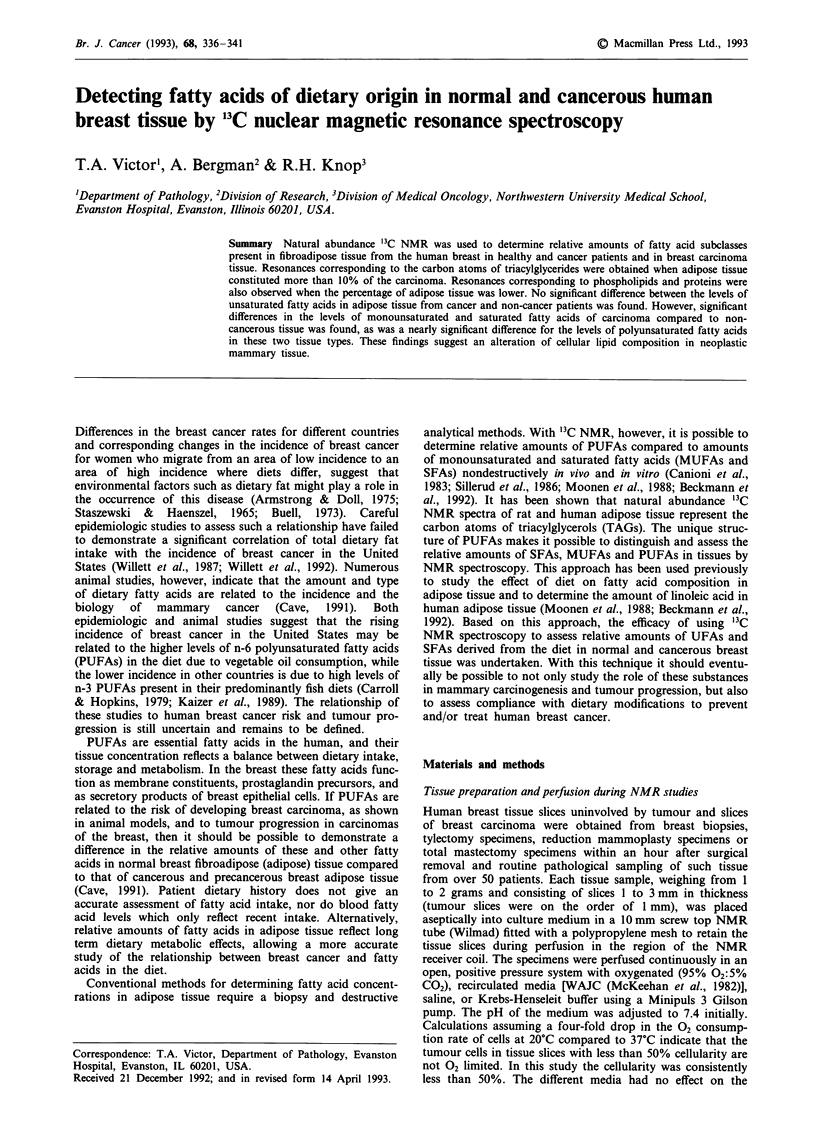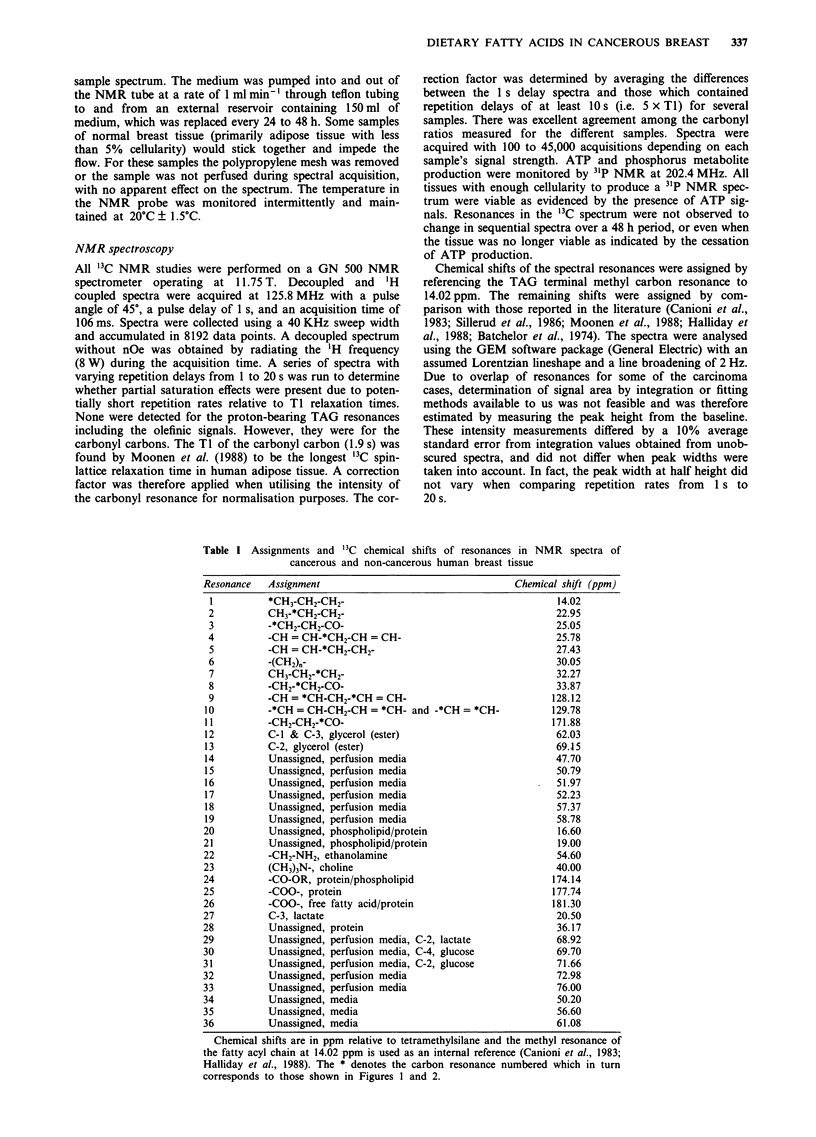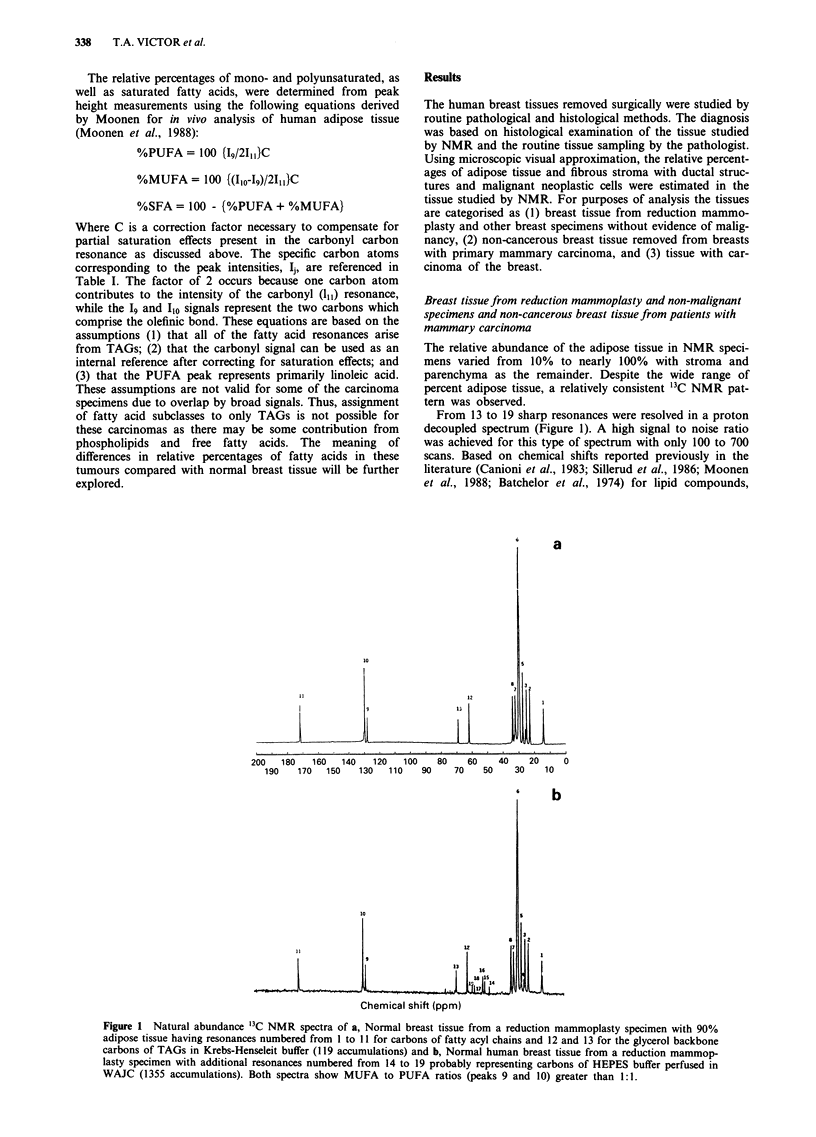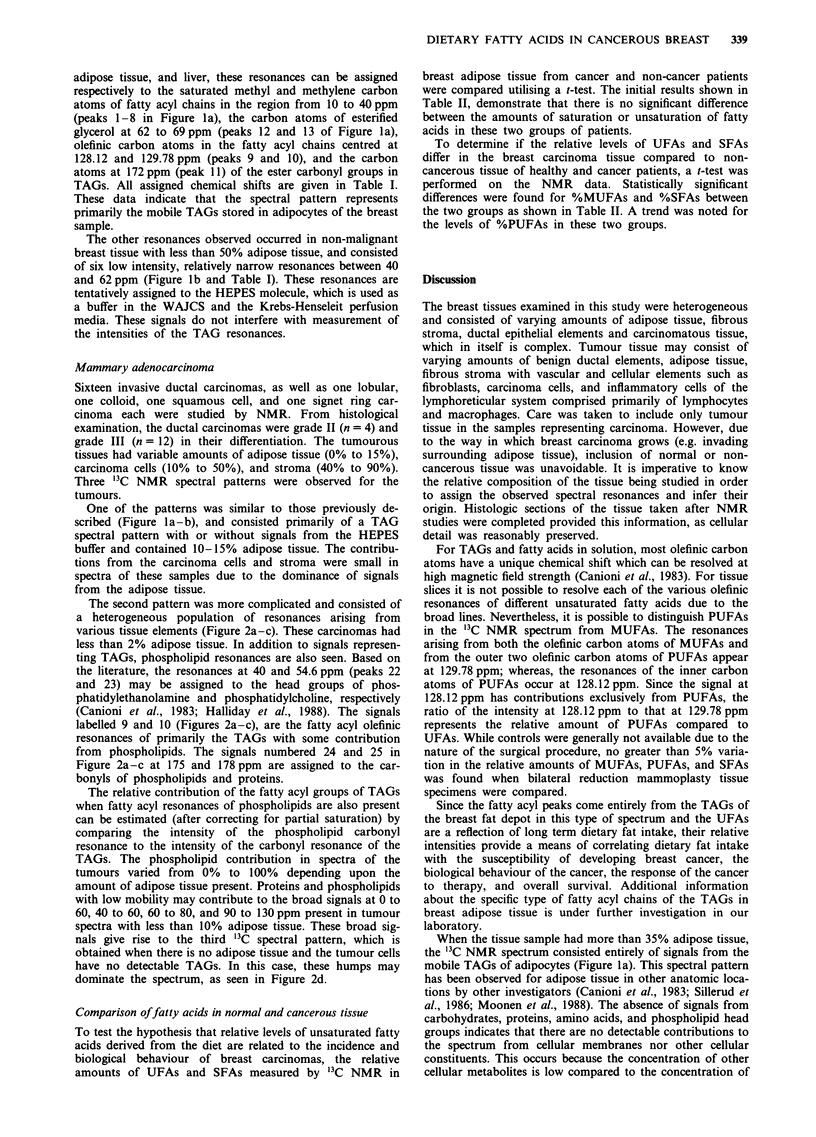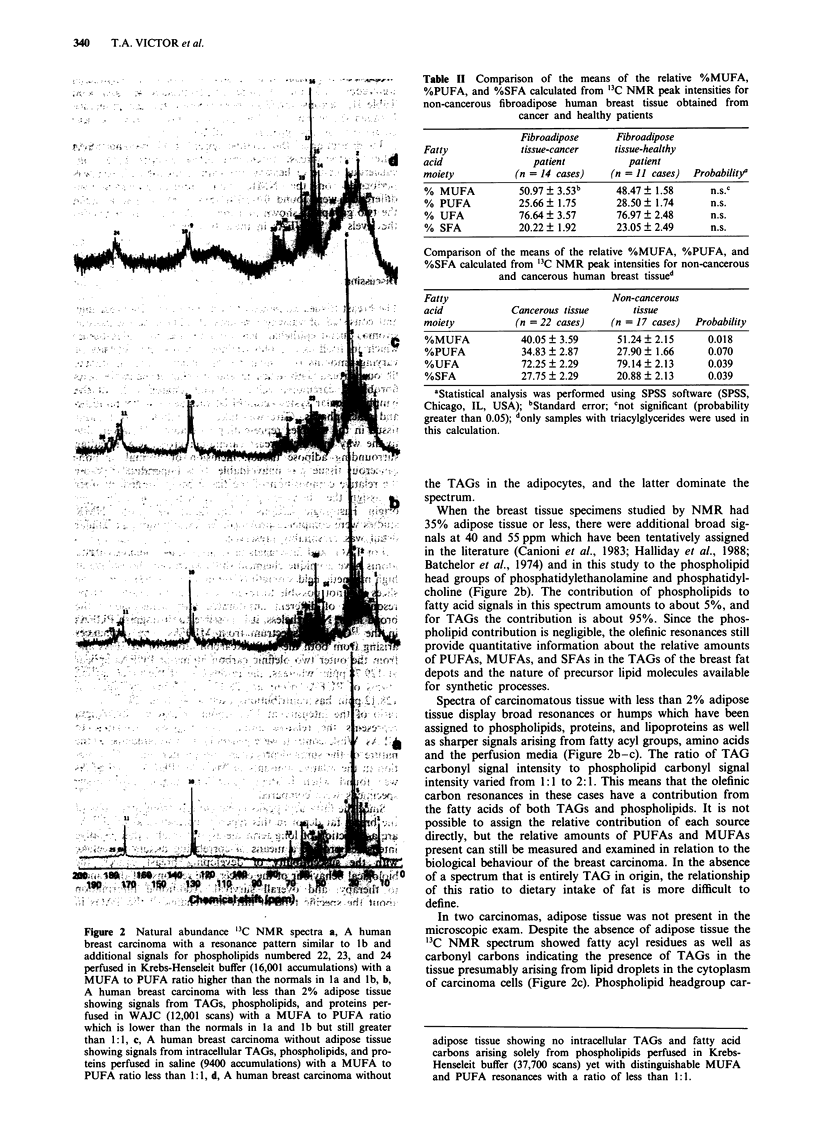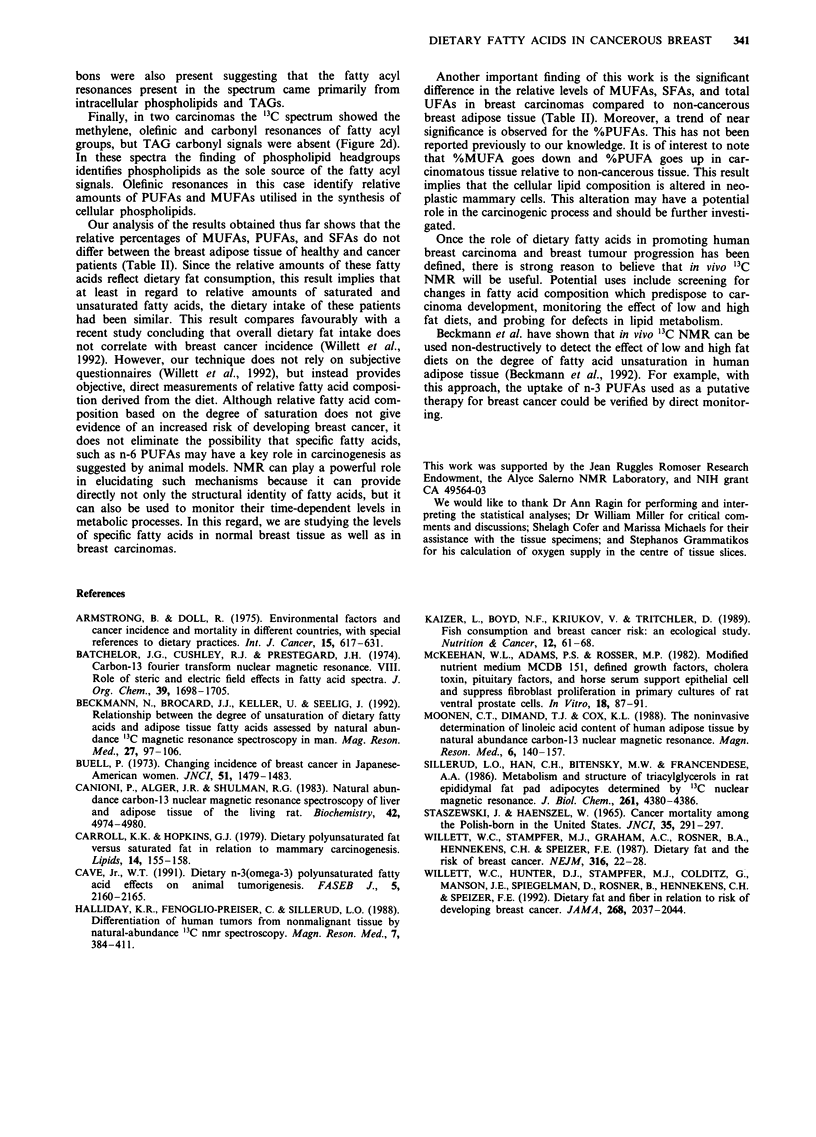# Detecting fatty acids of dietary origin in normal and cancerous human breast tissue by 13C nuclear magnetic resonance spectroscopy.

**DOI:** 10.1038/bjc.1993.337

**Published:** 1993-08

**Authors:** T. A. Victor, A. Bergman, R. H. Knop

**Affiliations:** Department of Pathology, Northwestern University Medical School, Evanston Hospital, Illinois 60201.

## Abstract

Natural abundance 13C NMR was used to determine relative amounts of fatty acid subclasses present in fibroadipose tissue from the human breast in healthy and cancer patients and in breast carcinoma tissue. Resonances corresponding to the carbon atoms of triacylglycerides were obtained when adipose tissue constituted more than 10% of the carcinoma. Resonances corresponding to phospholipids and proteins were also observed when the percentage of adipose tissue was lower. No significant difference between the levels of unsaturated fatty acids in adipose tissue from cancer and non-cancer patients was found. However, significant differences in the levels of monounsaturated and saturated fatty acids of carcinoma compared to non-cancerous tissue was found, as was a nearly significant difference for the levels of polyunsaturated fatty acids in these two tissue types. These findings suggest an alteration of cellular lipid composition in neoplastic mammary tissue.


Br. J. Cancer (1993), 68, 336-341 © Macmillan Press Ltd., 1993

### Detecting fatty acids of dietary origin in normal and cancerous human breast tissue by $^{13}$C nuclear magnetic resonance spectroscopy

T.A. Victor[1], A. Bergman[2] & R.H. Knop[3]

*[1]Department of Pathology, [2]Division of Research, [3]Division of Medical Oncology, Northwestern University Medical School, Evanston Hospital, Evanston, Illinois 60201, USA.*

**Summary** Natural abundance $^{13}$C NMR was used to determine relative amounts of fatty acid subclasses present in fibroadipose tissue from the human breast in healthy and cancer patients and in breast carcinoma tissue. Resonances corresponding to the carbon atoms of triacylglycerides were obtained when adipose tissue constituted more than 10% of the carcinoma. Resonances corresponding to phospholipids and proteins were also observed when the percentage of adipose tissue was lower. No significant difference between the levels of unsaturated fatty acids in adipose tissue from cancer and non-cancer patients was found. However, significant differences in the levels of monounsaturated and saturated fatty acids of carcinoma compared to non-cancerous tissue was found, as was a nearly significant difference for the levels of polyunsaturated fatty acids in these two tissue types. These findings suggest an alteration of cellular lipid composition in neoplastic mammary tissue.

Differences in the breast cancer rates for different countries and corresponding changes in the incidence of breast cancer for women who migrate from an area of low incidence to an area of high incidence where diets differ, suggest that environmental factors such as dietary fat might play a role in the occurrence of this disease (Armstrong & Doll, 1975; Staszewski & Haenszel, 1965; Buell, 1973). Careful epidemiologic studies to assess such a relationship have failed to demonstrate a significant correlation of total dietary fat intake with the incidence of breast cancer in the United States (Willett *et al.*, 1987; Willett *et al.*, 1992). Numerous animal studies, however, indicate that the amount and type of dietary fatty acids are related to the incidence and the biology of mammary cancer (Cave, 1991). Both epidemiologic and animal studies suggest that the rising incidence of breast cancer in the United States may be related to the higher levels of n-6 polyunsaturated fatty acids (PUFAs) in the diet due to vegetable oil consumption, while the lower incidence in other countries is due to high levels of n-3 PUFAs present in their predominantly fish diets (Carroll & Hopkins, 1979; Kaizer *et al.*, 1989). The relationship of these studies to human breast cancer risk and tumour progression is still uncertain and remains to be defined.

PUFAs are essential fatty acids in the human, and their tissue concentration reflects a balance between dietary intake, storage and metabolism. In the breast these fatty acids function as membrane constituents, prostaglandin precursors, and as secretory products of breast epithelial cells. If PUFAs are related to the risk of developing breast carcinoma, as shown in animal models, and to tumour progression in carcinomas of the breast, then it should be possible to demonstrate a difference in the relative amounts of these and other fatty acids in normal breast fibroadipose (adipose) tissue compared to that of cancerous and precancerous breast adipose tissue (Cave, 1991). Patient dietary history does not give an accurate assessment of fatty acid intake, nor do blood fatty acid levels which only reflect recent intake. Alternatively, relative amounts of fatty acids in adipose tissue reflect long term dietary metabolic effects, allowing a more accurate study of the relationship between breast cancer and fatty acids in the diet.

Conventional methods for determining fatty acid concentrations in adipose tissue require a biopsy and destructive analytical methods. With $^{13}$C NMR, however, it is possible to determine relative amounts of PUFAs compared to amounts of monounsaturated and saturated fatty acids (MUFAs and SFAs) nondestructively *in vivo* and *in vitro* (Canioni *et al.*, 1983; Sillerud *et al.*, 1986; Moonen *et al.*, 1988; Beckmann *et al.*, 1992). It has been shown that natural abundance $^{13}$C NMR spectra of rat and human adipose tissue represent the carbon atoms of triacylglycerols (TAGs). The unique structure of PUFAs makes it possible to distinguish and assess the relative amounts of SFAs, MUFAs and PUFAs in tissues by NMR spectroscopy. This approach has been used previously to study the effect of diet on fatty acid composition in adipose tissue and to determine the amount of linoleic acid in human adipose tissue (Moonen *et al.*, 1988; Beckmann *et al.*, 1992). Based on this approach, the efficacy of using $^{13}$C NMR spectroscopy to assess relative amounts of UFAs and SFAs derived from the diet in normal and cancerous breast tissue was undertaken. With this technique it should eventually be possible to not only study the role of these substances in mammary carcinogenesis and tumour progression, but also to assess compliance with dietary modifications to prevent and/or treat human breast cancer.

### Materials and methods

### *Tissue preparation and perfusion during NMR studies*

Human breast tissue slices uninvolved by tumour and slices of breast carcinoma were obtained from breast biopsies, tylectomy specimens, reduction mammoplasty specimens or total mastectomy specimens within an hour after surgical removal and routine pathological sampling of such tissue from over 50 patients. Each tissue sample, weighing from 1 to 2 grams and consisting of slices 1 to 3 mm in thickness (tumour slices were on the order of 1 mm), was placed aseptically into culture medium in a 10 mm screw top NMR tube (Wilmad) fitted with a polypropylene mesh to retain the tissue slices during perfusion in the region of the NMR receiver coil. The specimens were perfused continuously in an open, positive pressure system with oxygenated (95% $O_2$:5% $CO_2$), recirculated media [WAJC (McKeehan *et al.*, 1982)], saline, or Krebs-Henseleit buffer using a Minipuls 3 Gilson pump. The pH of the medium was adjusted to 7.4 initially. Calculations assuming a four-fold drop in the $O_2$ consumption rate of cells at 20°C compared to 37°C indicate that the tumour cells in tissue slices with less than 50% cellularity are not $O_2$ limited. In this study the cellularity was consistently less than 50%. The different media had no effect on the

Correspondence: T.A. Victor, Department of Pathology, Evanston Hospital, Evanston, IL 60201, USA.
Received 21 December 1992; and in revised form 14 April 1993.

sample spectrum. The medium was pumped into and out of the NMR tube at a rate of 1 ml $min^{-1}$ through teflon tubing to and from an external reservoir containing 150 ml of medium, which was replaced every 24 to 48 h. Some samples of normal breast tissue (primarily adipose tissue with less than 5% cellularity) would stick together and impede the flow. For these samples the polypropylene mesh was removed or the sample was not perfused during spectral acquisition, with no apparent effect on the spectrum. The temperature in the NMR probe was monitored intermittently and maintained at 20°C ± 1.5°C.

*NMR spectroscopy*

All $^{13}C$ NMR studies were performed on a GN 500 NMR spectrometer operating at 11.75 T. Decoupled and $^{1}H$ coupled spectra were acquired at 125.8 MHz with a pulse angle of 45°, a pulse delay of 1 s, and an acquisition time of 106 ms. Spectra were collected using a 40 KHz sweep width and accumulated in 8192 data points. A decoupled spectrum without nOe was obtained by radiating the $^{1}H$ frequency (8 W) during the acquisition time. A series of spectra with varying repetition delays from 1 to 20 s was run to determine whether partial saturation effects were present due to potentially short repetition rates relative to T1 relaxation times. None were detected for the proton-bearing TAG resonances including the olefinic signals. However, they were for the carbonyl carbons. The T1 of the carbonyl carbon (1.9 s) was found by Moonen *et al.* (1988) to be the longest $^{13}C$ spin-lattice relaxation time in human adipose tissue. A correction factor was therefore applied when utilising the intensity of the carbonyl resonance for normalisation purposes. The correction factor was determined by averaging the differences between the 1 s delay spectra and those which contained repetition delays of at least 10 s (i.e. 5 × T1) for several samples. There was excellent agreement among the carbonyl ratios measured for the different samples. Spectra were acquired with 100 to 45,000 acquisitions depending on each sample's signal strength. ATP and phosphorus metabolite production were monitored by $^{31}P$ NMR at 202.4 MHz. All tissues with enough cellularity to produce a $^{31}P$ NMR spectrum were viable as evidenced by the presence of ATP signals. Resonances in the $^{13}C$ spectrum were not observed to change in sequential spectra over a 48 h period, or even when the tissue was no longer viable as indicated by the cessation of ATP production.

Chemical shifts of the spectral resonances were assigned by referencing the TAG terminal methyl carbon resonance to 14.02 ppm. The remaining shifts were assigned by comparison with those reported in the literature (Canioni *et al.*, 1983; Sillerud *et al.*, 1986; Moonen *et al.*, 1988; Halliday *et al.*, 1988; Batchelor *et al.*, 1974). The spectra were analysed using the GEM software package (General Electric) with an assumed Lorentzian lineshape and a line broadening of 2 Hz. Due to overlap of resonances for some of the carcinoma cases, determination of signal area by integration or fitting methods available to us was not feasible and was therefore estimated by measuring the peak height from the baseline. These intensity measurements differed by a 10% average standard error from integration values obtained from unobscured spectra, and did not differ when peak widths were taken into account. In fact, the peak width at half height did not vary when comparing repetition rates from 1 s to 20 s.

**Table I** Assignments and $^{13}C$ chemical shifts of resonances in NMR spectra of cancerous and non-cancerous human breast tissue

| *Resonance* | *Assignment* | *Chemical shift (ppm)* |
|---|---|---|
| 1 | $*CH_3-CH_2-CH_2-$ | 14.02 |
| 2 | $CH_3-*CH_2-CH_2-$ | 22.95 |
| 3 | $-*CH_2-CH_2-CO-$ | 25.05 |
| 4 | $-CH = CH-*CH_2-CH = CH-$ | 25.78 |
| 5 | $-CH = CH-*CH_2-CH_2-$ | 27.43 |
| 6 | $-(CH_2)_n-$ | 30.05 |
| 7 | $CH_3-CH_2-*CH_2-$ | 32.27 |
| 8 | $-CH_2-*CH_2-CO-$ | 33.87 |
| 9 | $-CH = *CH-CH_2-*CH = CH-$ | 128.12 |
| 10 | $-*CH = CH-CH_2-CH = *CH-$ and $-*CH = *CH-$ | 129.78 |
| 11 | $-CH_2-CH_2-*CO-$ | 171.88 |
| 12 | C-1 & C-3, glycerol (ester) | 62.03 |
| 13 | C-2, glycerol (ester) | 69.15 |
| 14 | Unassigned, perfusion media | 47.70 |
| 15 | Unassigned, perfusion media | 50.79 |
| 16 | Unassigned, perfusion media | 51.97 |
| 17 | Unassigned, perfusion media | 52.23 |
| 18 | Unassigned, perfusion media | 57.37 |
| 19 | Unassigned, perfusion media | 58.78 |
| 20 | Unassigned, phospholipid/protein | 16.60 |
| 21 | Unassigned, phospholipid/protein | 19.00 |
| 22 | $-CH_2-NH_2$, ethanolamine | 54.60 |
| 23 | $(CH_3)_3N-$, choline | 40.00 |
| 24 | -CO-OR, protein/phospholipid | 174.14 |
| 25 | -COO-, protein | 177.74 |
| 26 | -COO-, free fatty acid/protein | 181.30 |
| 27 | C-3, lactate | 20.50 |
| 28 | Unassigned, protein | 36.17 |
| 29 | Unassigned, perfusion media, C-2, lactate | 68.92 |
| 30 | Unassigned, perfusion media, C-4, glucose | 69.70 |
| 31 | Unassigned, perfusion media, C-2, glucose | 71.66 |
| 32 | Unassigned, perfusion media | 72.98 |
| 33 | Unassigned, perfusion media | 76.00 |
| 34 | Unassigned, media | 50.20 |
| 35 | Unassigned, media | 56.60 |
| 36 | Unassigned, media | 61.08 |

Chemical shifts are in ppm relative to tetramethylsilane and the methyl resonance of the fatty acyl chain at 14.02 ppm is used as an internal reference (Canioni *et al.*, 1983; Halliday *et al.*, 1988). The * denotes the carbon resonance numbered which in turn corresponds to those shown in Figures 1 and 2.

The relative percentages of mono- and polyunsaturated, as well as saturated fatty acids, were determined from peak height measurements using the following equations derived by Moonen for *in vivo* analysis of human adipose tissue (Moonen *et al.*, 1988):

$$\%PUFA = 100 \{I_9/2I_{11}\}C$$

$$\%MUFA = 100 \{(I_{10}-I_9)/2I_{11}\}C$$

$$\%SFA = 100 - \{\%PUFA + \%MUFA\}$$

Where C is a correction factor necessary to compensate for partial saturation effects present in the carbonyl carbon resonance as discussed above. The specific carbon atoms corresponding to the peak intensities, $I_j$, are referenced in Table I. The factor of 2 occurs because one carbon atom contributes to the intensity of the carbonyl $(I_{11})$ resonance, while the $I_9$ and $I_{10}$ signals represent the two carbons which comprise the olefinic bond. These equations are based on the assumptions (1) that all of the fatty acid resonances arise from TAGs; (2) that the carbonyl signal can be used as an internal reference after correcting for saturation effects; and (3) that the PUFA peak represents primarily linoleic acid. These assumptions are not valid for some of the carcinoma specimens due to overlap by broad signals. Thus, assignment of fatty acid subclasses to only TAGs is not possible for these carcinomas as there may be some contribution from phospholipids and free fatty acids. The meaning of differences in relative percentages of fatty acids in these tumours compared with normal breast tissue will be further explored.

### Results

The human breast tissues removed surgically were studied by routine pathological and histological methods. The diagnosis was based on histological examination of the tissue studied by NMR and the routine tissue sampling by the pathologist. Using microscopic visual approximation, the relative percentages of adipose tissue and fibrous stroma with ductal structures and malignant neoplastic cells were estimated in the tissue studied by NMR. For purposes of analysis the tissues are categorised as (1) breast tissue from reduction mammoplasty and other breast specimens without evidence of malignancy, (2) non-cancerous breast tissue removed from breasts with primary mammary carcinoma, and (3) tissue with carcinoma of the breast.

### *Breast tissue from reduction mammoplasty and non-malignant specimens and non-cancerous breast tissue from patients with mammary carcinoma*

The relative abundance of the adipose tissue in NMR specimens varied from 10% to nearly 100% with stroma and parenchyma as the remainder. Despite the wide range of percent adipose tissue, a relatively consistent $^{13}C$ NMR pattern was observed.

From 13 to 19 sharp resonances were resolved in a proton decoupled spectrum (Figure 1). A high signal to noise ratio was achieved for this type of spectrum with only 100 to 700 scans. Based on chemical shifts reported previously in the literature (Canioni *et al.*, 1983; Sillerud *et al.*, 1986; Moonen *et al.*, 1988; Batchelor *et al.*, 1974) for lipid compounds,

**Figure 1** Natural abundance $^{13}C$ NMR spectra of **a**, Normal breast tissue from a reduction mammoplasty specimen with 90% adipose tissue having resonances numbered from 1 to 11 for carbons of fatty acyl chains and 12 and 13 for the glycerol backbone carbons of TAGs in Krebs-Henseleit buffer (119 accumulations) and **b**, Normal human breast tissue from a reduction mammoplasty specimen with additional resonances numbered from 14 to 19 probably representing carbons of HEPES buffer perfused in WAJC (1355 accumulations). Both spectra show MUFA to PUFA ratios (peaks 9 and 10) greater than 1:1.

adipose tissue, and liver, these resonances can be assigned respectively to the saturated methyl and methylene carbon atoms of fatty acyl chains in the region from 10 to 40 ppm (peaks 1–8 in Figure 1a), the carbon atoms of esterified glycerol at 62 to 69 ppm (peaks 12 and 13 of Figure 1a), olefinic carbon atoms in the fatty acyl chains centred at 128.12 and 129.78 ppm (peaks 9 and 10), and the carbon atoms at 172 ppm (peak 11) of the ester carbonyl groups in TAGs. All assigned chemical shifts are given in Table I. These data indicate that the spectral pattern represents primarily the mobile TAGs stored in adipocytes of the breast sample.

The other resonances observed occurred in non-malignant breast tissue with less than 50% adipose tissue, and consisted of six low intensity, relatively narrow resonances between 40 and 62 ppm (Figure 1b and Table I). These resonances are tentatively assigned to the HEPES molecule, which is used as a buffer in the WAJCS and the Krebs-Henseleit perfusion media. These signals do not interfere with measurement of the intensities of the TAG resonances.

### *Mammary adenocarcinoma*

Sixteen invasive ductal carcinomas, as well as one lobular, one colloid, one squamous cell, and one signet ring carcinoma each were studied by NMR. From histological examination, the ductal carcinomas were grade II ($n = 4$) and grade III ($n = 12$) in their differentiation. The tumourous tissues had variable amounts of adipose tissue (0% to 15%), carcinoma cells (10% to 50%), and stroma (40% to 90%). Three $^{13}C$ NMR spectral patterns were observed for the tumours.

One of the patterns was similar to those previously described (Figure 1a–b), and consisted primarily of a TAG spectral pattern with or without signals from the HEPES buffer and contained 10–15% adipose tissue. The contributions from the carcinoma cells and stroma were small in spectra of these samples due to the dominance of signals from the adipose tissue.

The second pattern was more complicated and consisted of a heterogeneous population of resonances arising from various tissue elements (Figure 2a–c). These carcinomas had less than 2% adipose tissue. In addition to signals representing TAGs, phospholipid resonances are also seen. Based on the literature, the resonances at 40 and 54.6 ppm (peaks 22 and 23) may be assigned to the head groups of phosphatidylethanolamine and phosphatidylcholine, respectively (Canioni *et al.*, 1983; Halliday *et al.*, 1988). The signals labelled 9 and 10 (Figures 2a–c), are the fatty acyl olefinic resonances of primarily the TAGs with some contribution from phospholipids. The signals numbered 24 and 25 in Figure 2a–c at 175 and 178 ppm are assigned to the carbonyls of phospholipids and proteins.

The relative contribution of the fatty acyl groups of TAGs when fatty acyl resonances of phospholipids are also present can be estimated (after correcting for partial saturation) by comparing the intensity of the phospholipid carbonyl resonance to the intensity of the carbonyl resonance of the TAGs. The phospholipid contribution in spectra of the tumours varied from 0% to 100% depending upon the amount of adipose tissue present. Proteins and phospholipids with low mobility may contribute to the broad signals at 0 to 60, 40 to 60, 60 to 80, and 90 to 130 ppm present in tumour spectra with less than 10% adipose tissue. These broad signals give rise to the third $^{13}C$ spectral pattern, which is obtained when there is no adipose tissue and the tumour cells have no detectable TAGs. In this case, these humps may dominate the spectrum, as seen in Figure 2d.

### *Comparison of fatty acids in normal and cancerous tissue*

To test the hypothesis that relative levels of unsaturated fatty acids derived from the diet are related to the incidence and biological behaviour of breast carcinomas, the relative amounts of UFAs and SFAs measured by $^{13}C$ NMR in breast adipose tissue from cancer and non-cancer patients were compared utilising a *t*-test. The initial results shown in Table II, demonstrate that there is no significant difference between the amounts of saturation or unsaturation of fatty acids in these two groups of patients.

To determine if the relative levels of UFAs and SFAs differ in the breast carcinoma tissue compared to non-cancerous tissue of healthy and cancer patients, a *t*-test was performed on the NMR data. Statistically significant differences were found for %MUFAs and %SFAs between the two groups as shown in Table II. A trend was noted for the levels of %PUFAs in these two groups.

### Discussion

The breast tissues examined in this study were heterogeneous and consisted of varying amounts of adipose tissue, fibrous stroma, ductal epithelial elements and carcinomatous tissue, which in itself is complex. Tumour tissue may consist of varying amounts of benign ductal elements, adipose tissue, fibrous stroma with vascular and cellular elements such as fibroblasts, carcinoma cells, and inflammatory cells of the lymphoreticular system comprised primarily of lymphocytes and macrophages. Care was taken to include only tumour tissue in the samples representing carcinoma. However, due to the way in which breast carcinoma grows (e.g. invading surrounding adipose tissue), inclusion of normal or non-cancerous tissue was unavoidable. It is imperative to know the relative composition of the tissue being studied in order to assign the observed spectral resonances and infer their origin. Histologic sections of the tissue taken after NMR studies were completed provided this information, as cellular detail was reasonably preserved.

For TAGs and fatty acids in solution, most olefinic carbon atoms have a unique chemical shift which can be resolved at high magnetic field strength (Canioni *et al.*, 1983). For tissue slices it is not possible to resolve each of the various olefinic resonances of different unsaturated fatty acids due to the broad lines. Nevertheless, it is possible to distinguish PUFAs in the $^{13}C$ NMR spectrum from MUFAs. The resonances arising from both the olefinic carbon atoms of MUFAs and from the outer two olefinic carbon atoms of PUFAs appear at 129.78 ppm; whereas, the resonances of the inner carbon atoms of PUFAs occur at 128.12 ppm. Since the signal at 128.12 ppm has contributions exclusively from PUFAs, the ratio of the intensity at 128.12 ppm to that at 129.78 ppm represents the relative amount of PUFAs compared to UFAs. While controls were generally not available due to the nature of the surgical procedure, no greater than 5% variation in the relative amounts of MUFAs, PUFAs, and SFAs was found when bilateral reduction mammoplasty tissue specimens were compared.

Since the fatty acyl peaks come entirely from the TAGs of the breast fat depot in this type of spectrum and the UFAs are a reflection of long term dietary fat intake, their relative intensities provide a means of correlating dietary fat intake with the susceptibility of developing breast cancer, the biological behaviour of the cancer, the response of the cancer to therapy, and overall survival. Additional information about the specific type of fatty acyl chains of the TAGs in breast adipose tissue is under further investigation in our laboratory.

When the tissue sample had more than 35% adipose tissue, the $^{13}C$ NMR spectrum consisted entirely of signals from the mobile TAGs of adipocytes (Figure 1a). This spectral pattern has been observed for adipose tissue in other anatomic locations by other investigators (Canioni *et al.*, 1983; Sillerud *et al.*, 1986; Moonen *et al.*, 1988). The absence of signals from carbohydrates, proteins, amino acids, and phospholipid head groups indicates that there are no detectable contributions to the spectrum from cellular membranes nor other cellular constituents. This occurs because the concentration of other cellular metabolites is low compared to the concentration of

**Figure 2** Natural abundance $^{13}C$ NMR spectra **a**, A human breast carcinoma with a resonance pattern similar to 1b and additional signals for phospholipids numbered 22, 23, and 24 perfused in Krebs-Henseleit buffer (16,001 accumulations) with a MUFA to PUFA ratio higher than the normals in 1a and 1b, **b**, A human breast carcinoma with less than 2% adipose tissue showing signals from TAGs, phospholipids, and proteins perfused in WAJC (12,001 scans) with a MUFA to PUFA ratio which is lower than the normals in 1a and 1b but still greater than 1:1, **c**, A human breast carcinoma without adipose tissue showing signals from intracellular TAGs, phospholipids, and proteins perfused in saline (9400 accumulations) with a MUFA to PUFA ratio less than 1:1, **d**, A human breast carcinoma without adipose tissue showing no intracellular TAGs and fatty acid carbons arising solely from phospholipids perfused in Krebs-Henseleit buffer (37,700 scans) yet with distinguishable MUFA and PUFA resonances with a ratio of less than 1:1.

**Table II** Comparison of the means of the relative %MUFA, %PUFA, and %SFA calculated from $^{13}C$ NMR peak intensities for non-cancerous fibroadipose human breast tissue obtained from cancer and healthy patients

| *Fatty acid moiety* | *Fibroadipose tissue-cancer patient (n = 14 cases)* | *Fibroadipose tissue-healthy patient (n = 11 cases)* | *Probability*[a] |
|---|---|---|---|
| % MUFA | 50.97 ± 3.53[b] | 48.47 ± 1.58 | n.s.[c] |
| % PUFA | 25.66 ± 1.75 | 28.50 ± 1.74 | n.s. |
| % UFA | 76.64 ± 3.57 | 76.97 ± 2.48 | n.s. |
| % SFA | 20.22 ± 1.92 | 23.05 ± 2.49 | n.s. |

Comparison of the means of the relative %MUFA, %PUFA, and %SFA calculated from $^{13}C$ NMR peak intensities for non-cancerous and cancerous human breast tissue[d]

| *Fatty acid moiety* | *Cancerous tissue (n = 22 cases)* | *Non-cancerous tissue (n = 17 cases)* | *Probability* |
|---|---|---|---|
| %MUFA | 40.05 ± 3.59 | 51.24 ± 2.15 | 0.018 |
| %PUFA | 34.83 ± 2.87 | 27.90 ± 1.66 | 0.070 |
| %UFA | 72.25 ± 2.29 | 79.14 ± 2.13 | 0.039 |
| %SFA | 27.75 ± 2.29 | 20.88 ± 2.13 | 0.039 |

[a]Statistical analysis was performed using SPSS software (SPSS, Chicago, IL, USA); [b]Standard error; [c]not significant (probability greater than 0.05); [d]only samples with triacylglycerides were used in this calculation.

the TAGs in the adipocytes, and the latter dominate the spectrum.

When the breast tissue specimens studied by NMR had 35% adipose tissue or less, there were additional broad signals at 40 and 55 ppm which have been tentatively assigned in the literature (Canioni *et al.*, 1983; Halliday *et al.*, 1988; Batchelor *et al.*, 1974) and in this study to the phospholipid head groups of phosphatidylethanolamine and phosphatidylcholine (Figure 2b). The contribution of phospholipids to fatty acid signals in this spectrum amounts to about 5%, and for TAGs the contribution is about 95%. Since the phospholipid contribution is negligible, the olefinic resonances still provide quantitative information about the relative amounts of PUFAs, MUFAs, and SFAs in the TAGs of the breast fat depots and the nature of precursor lipid molecules available for synthetic processes.

Spectra of carcinomatous tissue with less than 2% adipose tissue display broad resonances or humps which have been assigned to phospholipids, proteins, and lipoproteins as well as sharper signals arising from fatty acyl groups, amino acids and the perfusion media (Figure 2b–c). The ratio of TAG carbonyl signal intensity to phospholipid carbonyl signal intensity varied from 1:1 to 2:1. This means that the olefinic carbon resonances in these cases have a contribution from the fatty acids of both TAGs and phospholipids. It is not possible to assign the relative contribution of each source directly, but the relative amounts of PUFAs and MUFAs present can still be measured and examined in relation to the biological behaviour of the breast carcinoma. In the absence of a spectrum that is entirely TAG in origin, the relationship of this ratio to dietary intake of fat is more difficult to define.

In two carcinomas, adipose tissue was not present in the microscopic exam. Despite the absence of adipose tissue the $^{13}C$ NMR spectrum showed fatty acyl residues as well as carbonyl carbons indicating the presence of TAGs in the tissue presumably arising from lipid droplets in the cytoplasm of carcinoma cells (Figure 2c). Phospholipid headgroup carbons were also present suggesting that the fatty acyl resonances present in the spectrum came primarily from intracellular phospholipids and TAGs.

Finally, in two carcinomas the $^{13}C$ spectrum showed the methylene, olefinic and carbonyl resonances of fatty acyl groups, but TAG carbonyl signals were absent (Figure 2d). In these spectra the finding of phospholipid headgroups identifies phospholipids as the sole source of the fatty acyl signals. Olefinic resonances in this case identify relative amounts of PUFAs and MUFAs utilised in the synthesis of cellular phospholipids.

Our analysis of the results obtained thus far shows that the relative percentages of MUFAs, PUFAs, and SFAs do not differ between the breast adipose tissue of healthy and cancer patients (Table II). Since the relative amounts of these fatty acids reflect dietary fat consumption, this result implies that at least in regard to relative amounts of saturated and unsaturated fatty acids, the dietary intake of these patients had been similar. This result compares favourably with a recent study concluding that overall dietary fat intake does not correlate with breast cancer incidence (Willett *et al.*, 1992). However, our technique does not rely on subjective questionnaires (Willett *et al.*, 1992), but instead provides objective, direct measurements of relative fatty acid composition derived from the diet. Although relative fatty acid composition based on the degree of saturation does not give evidence of an increased risk of developing breast cancer, it does not eliminate the possibility that specific fatty acids, such as n-6 PUFAs may have a key role in carcinogenesis as suggested by animal models. NMR can play a powerful role in elucidating such mechanisms because it can provide directly not only the structural identity of fatty acids, but it can also be used to monitor their time-dependent levels in metabolic processes. In this regard, we are studying the levels of specific fatty acids in normal breast tissue as well as in breast carcinomas.

Another important finding of this work is the significant difference in the relative levels of MUFAs, SFAs, and total UFAs in breast carcinomas compared to non-cancerous breast adipose tissue (Table II). Moreover, a trend of near significance is observed for the %PUFAs. This has not been reported previously to our knowledge. It is of interest to note that %MUFA goes down and %PUFA goes up in carcinomatous tissue relative to non-cancerous tissue. This result implies that the cellular lipid composition is altered in neoplastic mammary cells. This alteration may have a potential role in the carcinogenic process and should be further investigated.

Once the role of dietary fatty acids in promoting human breast carcinoma and breast tumour progression has been defined, there is strong reason to believe that *in vivo* $^{13}C$ NMR will be useful. Potential uses include screening for changes in fatty acid composition which predispose to carcinoma development, monitoring the effect of low and high fat diets, and probing for defects in lipid metabolism.

Beckmann *et al.* have shown that *in vivo* $^{13}C$ NMR can be used non-destructively to detect the effect of low and high fat diets on the degree of fatty acid unsaturation in human adipose tissue (Beckmann *et al.*, 1992). For example, with this approach, the uptake of n-3 PUFAs used as a putative therapy for breast cancer could be verified by direct monitoring.

This work was supported by the Jean Ruggles Romoser Research Endowment, the Alyce Salerno NMR Laboratory, and NIH grant CA 49564-03

We would like to thank Dr Ann Ragin for performing and interpreting the statistical analyses; Dr William Miller for critical comments and discussions; Shelagh Cofer and Marissa Michaels for their assistance with the tissue specimens; and Stephanos Grammatikos for his calculation of oxygen supply in the centre of tissue slices.

### References

ARMSTRONG, B. & DOLL, R. (1975). Environmental factors and cancer incidence and mortality in different countries, with special references to dietary practices. *Int. J. Cancer*, **15**, 617–631.

BATCHELOR, J.G., CUSHLEY, R.J. & PRESTEGARD, J.H. (1974). Carbon-13 fourier transform nuclear magnetic resonance. VIII. Role of steric and electric field effects in fatty acid spectra. *J. Org. Chem.*, **39**, 1698–1705.

BECKMANN, N., BROCARD, J.J., KELLER, U. & SEELIG, J. (1992). Relationship between the degree of unsaturation of dietary fatty acids and adipose tissue fatty acids assessed by natural abundance $^{13}C$ magnetic resonance spectroscopy in man. *Mag. Reson. Med.*, **27**, 97–106.

BUELL, P. (1973). Changing incidence of breast cancer in Japanese-American women. *JNCI*, **51**, 1479–1483.

CANIONI, P., ALGER, J.R. & SHULMAN, R.G. (1983). Natural abundance carbon-13 nuclear magnetic resonance spectroscopy of liver and adipose tissue of the living rat. *Biochemistry*, **42**, 4974–4980.

CARROLL, K.K. & HOPKINS, G.J. (1979). Dietary polyunsaturated fat versus saturated fat in relation to mammary carcinogenesis. *Lipids*, **14**, 155–158.

CAVE, Jr., W.T. (1991). Dietary n-3(omega-3) polyunsaturated fatty acid effects on animal tumorigenesis. *FASEB J.*, **5**, 2160–2165.

HALLIDAY, K.R., FENOGLIO-PREISER, C. & SILLERUD, L.O. (1988). Differentiation of human tumors from nonmalignant tissue by natural-abundance $^{13}C$ nmr spectroscopy. *Magn. Reson. Med.*, **7**, 384–411.

KAIZER, L., BOYD, N.F., KRIUKOV, V. & TRITCHLER, D. (1989). Fish consumption and breast cancer risk: an ecological study. *Nutrition & Cancer*, **12**, 61–68.

MCKEEHAN, W.L., ADAMS, P.S. & ROSSER, M.P. (1982). Modified nutrient medium MCDB 151, defined growth factors, cholera toxin, pituitary factors, and horse serum support epithelial cell and suppress fibroblast proliferation in primary cultures of rat ventral prostate cells. *In Vitro*, **18**, 87–91.

MOONEN, C.T., DIMAND, T.J. & COX, K.L. (1988). The noninvasive determination of linoleic acid content of human adipose tissue by natural abundance carbon-13 nuclear magnetic resonance. *Magn. Reson. Med.*, **6**, 140–157.

SILLERUD, L.O., HAN, C.H., BITENSKY, M.W. & FRANCENDESE, A.A. (1986). Metabolism and structure of triacylglycerols in rat epididymal fat pad adipocytes determined by $^{13}C$ nuclear magnetic resonance. *J. Biol. Chem.*, **261**, 4380–4386.

STASZEWSKI, J. & HAENSZEL, W. (1965). Cancer mortality among the Polish-born in the United States. *JNCI*, **35**, 291–297.

WILLETT, W.C., STAMPFER, M.J., GRAHAM, A.C., ROSNER, B.A., HENNEKENS, C.H. & SPEIZER, F.E. (1987). Dietary fat and the risk of breast cancer. *NEJM*, **316**, 22–28.

WILLETT, W.C., HUNTER, D.J., STAMPFER, M.J., COLDITZ, G., MANSON, J.E., SPIEGELMAN, D., ROSNER, B., HENNEKENS, C.H. & SPEIZER, F.E. (1992). Dietary fat and fiber in relation to risk of developing breast cancer. *JAMA*, **268**, 2037–2044.